# A Shipping Container-Based Sterile Processing Unit for Low Resources Settings

**DOI:** 10.1371/journal.pone.0149624

**Published:** 2016-03-23

**Authors:** Jean Boubour, Katherine Jenson, Hannah Richter, Josiah Yarbrough, Z. Maria Oden, Douglas A. Schuler

**Affiliations:** 1 Association Soleil-Vapeur, Evreux, France; 2 Jones Graduate School of Business, Rice University, Houston, Texas, United States of America; 3 Brown School of Engineering, Department of Mechanical Engineering, Rice University, Houston, Texas, United States of America; 4 Brown School of Engineering, Department of Chemical Engineering, Rice University, Houston, Texas, United States of America; 5 Brown School of Engineering, Department of Bioengineering and Rice 360 Institute of Global Health, Rice University, Houston, Texas, United States of America; 6 Jones Graduate School of Business and Rice 360 Institute of Global Health, Rice University, Houston, Texas, United States of America; FDA, UNITED STATES

## Abstract

Deficiencies in the sterile processing of medical instruments contribute to poor outcomes for patients, such as surgical site infections, longer hospital stays, and deaths. In low resources settings, such as some rural and semi-rural areas and secondary and tertiary cities of developing countries, deficiencies in sterile processing are accentuated due to the lack of access to sterilization equipment, improperly maintained and malfunctioning equipment, lack of power to operate equipment, poor protocols, and inadequate quality control over inventory. Inspired by our sterile processing fieldwork at a district hospital in Sierra Leone in 2013, we built an autonomous, shipping-container-based sterile processing unit to address these deficiencies. The sterile processing unit, dubbed “the sterile box,” is a full suite capable of handling instruments from the moment they leave the operating room to the point they are sterile and ready to be reused for the next surgery. The sterile processing unit is self-sufficient in power and water and features an intake for contaminated instruments, decontamination, sterilization via non-electric steam sterilizers, and secure inventory storage. To validate efficacy, we ran tests of decontamination and sterilization performance. Results of 61 trials validate convincingly that our sterile processing unit achieves satisfactory outcomes for decontamination and sterilization and as such holds promise to support healthcare facilities in low resources settings.

## Introduction

Surgical site infections, one form of healthcare associated infections, [1] are the leading source of infection to patients in healthcare facilities in low resources settings with about one-third of surgical patients getting infected, a rate nine times greater than in developed countries. [2] The use of medical instruments contaminated with microorganisms (bioburden, such as tissue and blood remaining from a surgical procedure) that have not been properly cleaned and sterilized directly contribute to surgical site infections in other patients. Sterile processing aims at breaking this cycle. Sterile processing entails the collection and cleaning of contaminated medical instruments, preparation and packaging of such instruments for sterilization, sterilization via a sterilizer (i.e., autoclave), and the secure storage of such processed instruments. When properly conducted, sterile processing procedures reduce the likelihood that instruments act as vehicles for microorganisms to travel from one patient to another patient or to healthcare workers and can reduce significantly the chance that instruments lead to surgical site infections. [3]

Modern healthcare facilities attempt to minimize this risk through a rigorous approach to sterile processing, using equipment, layout, infrastructure, protocols, and training. Typical equipment in a sterile processing unit are instrument washers, vacuum autoclaves, electric washers (i.e., for endoscopes), gas chambers, and storage cabinets. The physical layout separates (oftentimes in different rooms) decontamination from sterilization and storage of sterilized instruments. Other features of a modern sterile processing facility are HVAC systems to minimize airborne contamination, treated water for decontamination and rinsing, and training and operating protocols to assure quality control. The equipment and infrastructure have high upfront and operating costs and considerable energy and water requirements. For example, per the authors’ correspondence in June 2015 with company representatives, a Getinge Model 422HC (gravity) steam sterilizer appropriate for a small hospital costs just over $30,000 USD and consumes 0.78kw of power and 285 gallons of water during its 2-hour cycle.

Because of the high up-front capital and operating costs and the uncertainty over the availability of power, healthcare facilities in low resources settings mostly use other sterile processing options. Instead of using electricity-driven mechanical washers, a health worker typically decontaminates instruments by hand by rinsing them in water, soaking in solutions (i.e., bleach, glutaraldehyde, dialdehyde, etc.), and scrubbing with nylon brushes. Under the best circumstances, sterilization is typically performed using non-electric steam sterilizers powered by gas with a burner although electric-powered steam sterilizers are sometimes used. Other methods, such as nitrogen dioxide sterilization, have been technically demonstrated [4] but are not in widespread use. However, due to inoperable equipment, lack of reliable power (i.e., gas or electricity [5]), and other factors, [6] sterilization oftentimes is not performed at all or is conducted in a technically inadequate way. Additionally, many healthcare facilities do not have a physical layout that satisfactorily separates contaminated from sterile medical instruments.

The inspiration for our sterile processing unit comes from observing many sterile processing deficiencies during our installation of a solar-powered autoclave [7] at a district hospital in Sierra Leone in 2013. The hospital’s sterile processing room did not segregate decontamination from sterilization and storage. Several of the sterilizers were in disrepair. Quality controls were not practiced. From observation, it appeared that the physical layout, equipment, and protocols most likely failed to achieve sterile outcomes. The sterile processing unit we designed aims to remedy these problems.

### Description of the sterile processing unit, “the sterile box”

Here, we briefly describe the sterile processing unit that we have designed and constructed for low resources settings (technical details appear in S1 Appendix).

**Fig 1:**
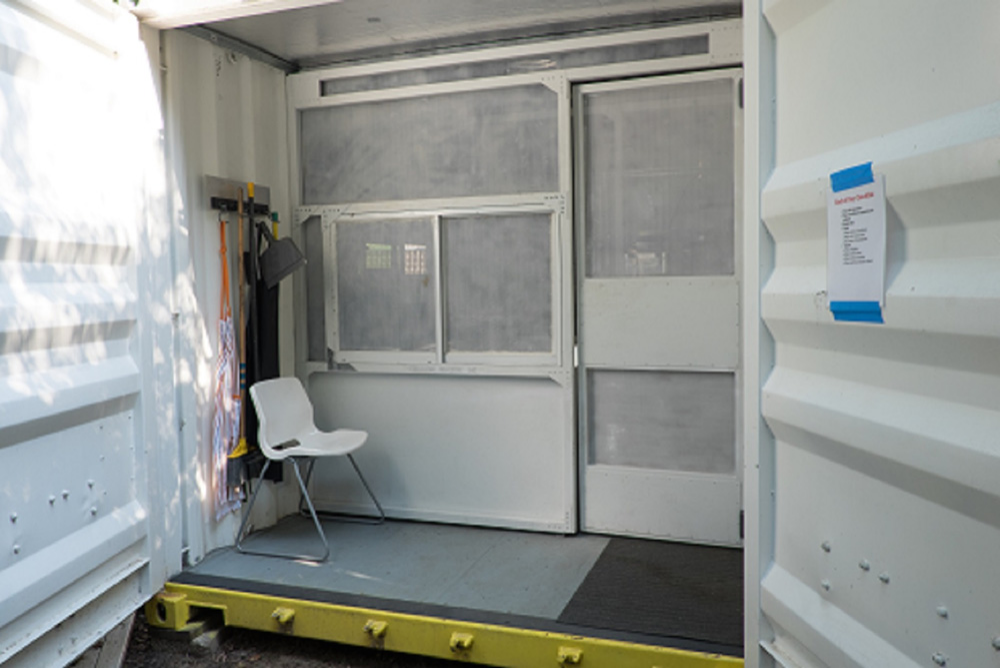
Foyer to receive and dispense instruments.

A standard twenty-foot (6.1m) steel shipping container houses our sterile processing unit. Inside the unit, a small foyer separates the sterile processing operations (described below) from environmental elements and also from outsiders. This area has a small window that allows staff from an affiliated hospital or healthcare center to pass soiled instruments to and receive sterile instruments from the sterile processing staff working inside the container (Fig 1). Four areas lay further inside: decontamination, partitioned from the other areas by a half wall, preparation, sterilization, and drying and storage.

**Fig 2:**
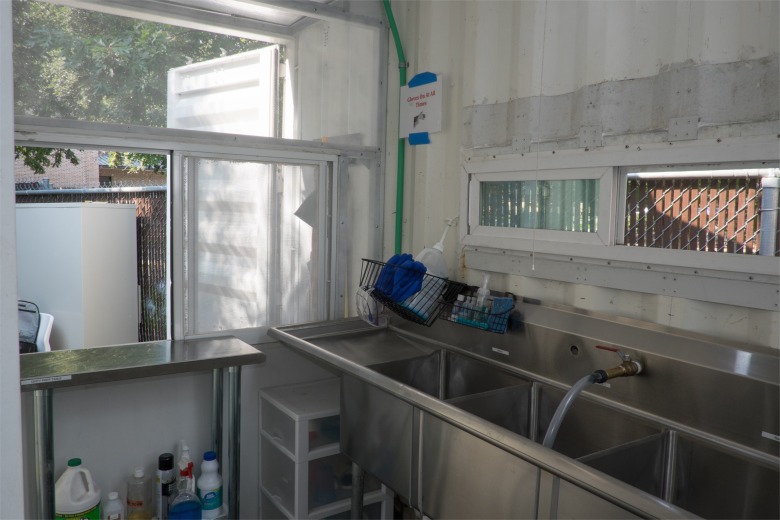
Sink in the decontamination area.

**Fig 3:**
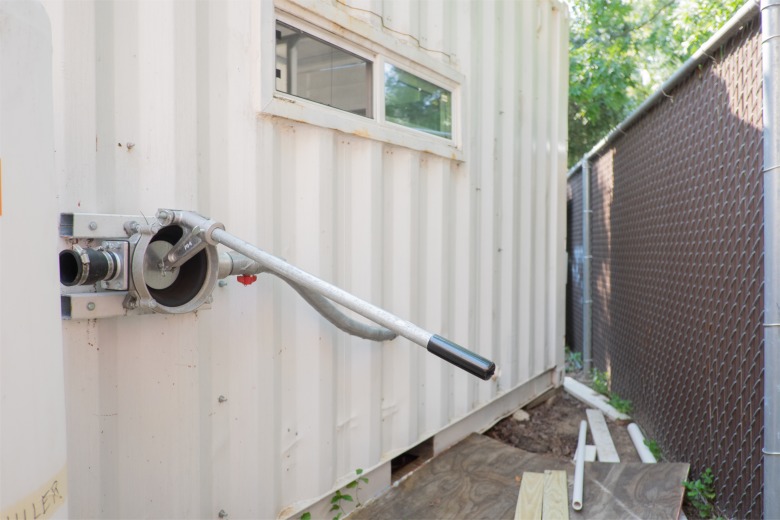
Water tank and hand-powered pump.

In the decontamination area, the staff performs decontamination via a three-basin sink as follows: the first sink is used to remove gross debris from the instruments; the second sink is used to soak instruments in enzymatic detergent followed by scrubbing with nylon brushes; and the third sink is used for final rinse (Fig 2). Water flows to the sink from a system of two tanks joined by tubes: a 55 gallon (208 l) receiving tank on the ground with a hand-powered diaphragm pump (Fig 3) that pumps water to a 50 gallon (189 l) tank located on the roof. At the sink, the staff controls a ball valve to bring water from the upper tank into the sink through tubing at a maximum flow rate of 22.2 liters/minute.

**Fig 4:**
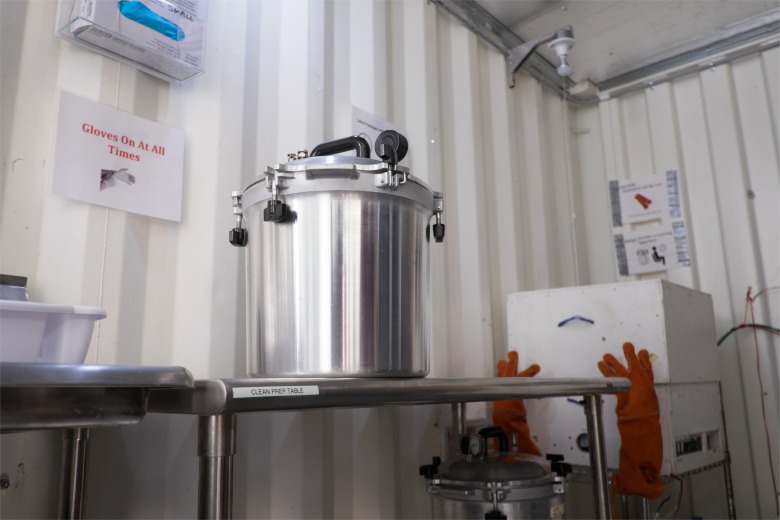
Non-electric steam sterilizer resting upon the preparation table.

A stainless steel work table occupies the preparation area (Fig 4).

**Fig 5:**
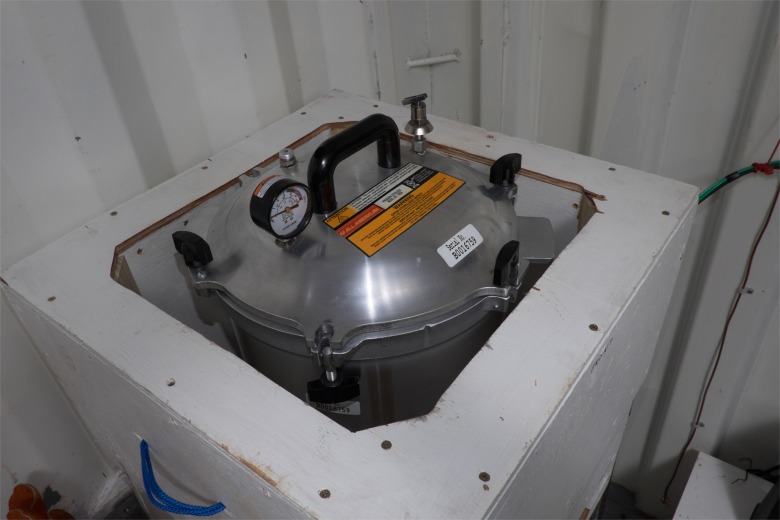
Sterilizer sitting on the electric hotplate.

**Fig 6:**
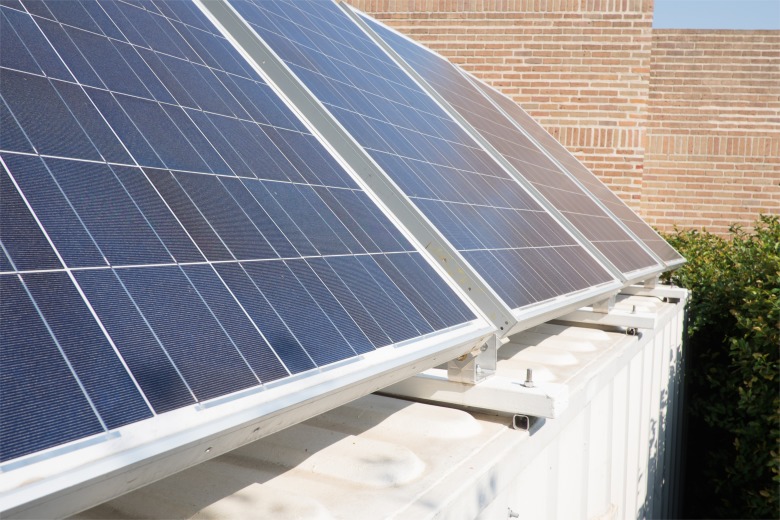
Solar PV panels mounted on container’s roof.

**Fig 7:**
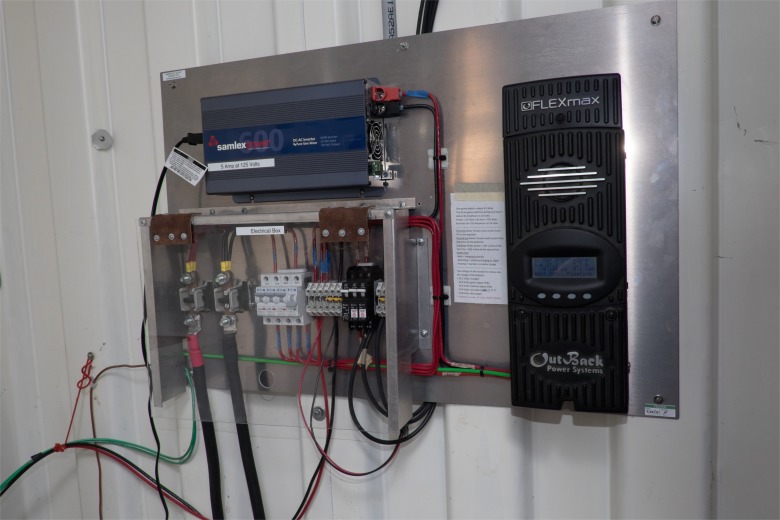
Electrical control plate inside of container.

In the sterilization area, a non-electric, gravity steam sterilizer (WAFCO 1925X), heated by a 750w electric hotplate that we constructed (Fig 5), sterilizes the instruments. Electricity comes from two 12V batteries joined into a 24V storage unit that is charged by a solar photovoltaic (PV) installation (four 230w panels for 920w or 0.92kw) mounted on the container’s roof (Fig 6). An Outback Power Systems Controller mounted inside the container regulates the flow of electricity (Fig 7).

**Fig 8:**
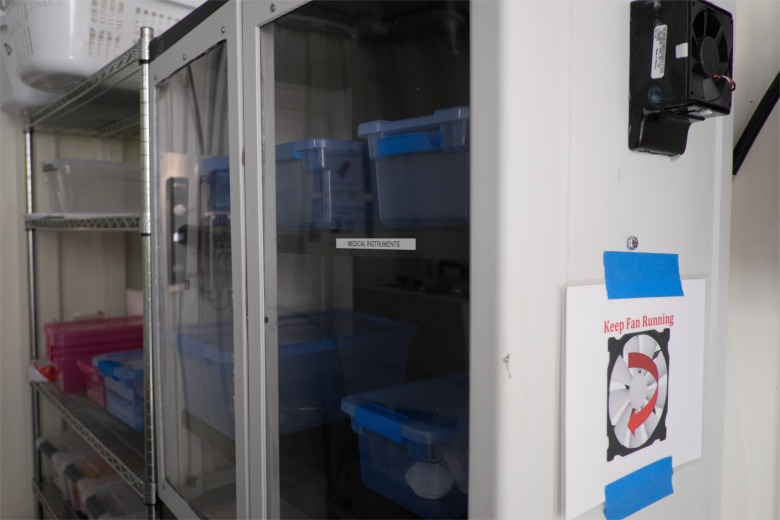
Racks and cabinet for the drying and storage of sterilized instruments.

In the drying and storage area, wire racks are used for the instruments to cool after sterilization and a storage cabinet (with plexiglas door) for the instruments to be held until requested by the healthcare facility’s staff (Fig 8).

The container’s usability is enhanced by several additional features. Daylight is provided by two windows and work in the dark is enabled by three 5w DC light bulbs powered by the solar PV electrical system. Ambient light is maximized by white paint on the interior walls. Air flow is facilitated by window screens, three floor level air vents, a mesh across our entire outside door opening (approximately 2.5m x 2.5m), and two wind-powered turbine fans through the ceiling. Temperatures inside the box are minimized by the use of radiant barrier insulation and reflective paint on the outer surfaces of the container. Functional outlets (for running small appliances such as a fan and charging cell phones and laptop computers) are supplied by a 600w inverter connected to the electrical system.

## Materials and Methods

To validate the performance of our sterile processing unit, we designed an experiment to test the (1) decontamination and (2) sterilization processes. Between May 27, 2015 and August 17, 2015, we conducted 61 trials of the sterile processing unit. Table 1 summarizes the measures, measurement tools, and the criteria for judging the test results.

**Table 1:** Decontamination and Sterilization Measures and Test Criteria.

Area	Measure	Measurement Tool	Criteria for “Pass” and “Fail”
Decontamination	ATP	ATP Bioluminescence Device	A swab touches the medical instruments’ surface and is inserted into a tube that in turn is inserted into the ATP device. ATP device reads relative light units. Readings at or below 45 (“Pass”) indicate the surface is considered clean for surgical instruments. Readings greater than 45 (“Fail”) indicate the surface is considered dirty.^a^
Sterilization	Temperature inside the sterilizer and Time of run	Temperature Gauge on Sterilizer and Electronic Timer	Proper exposure to steam, pressure, and time is known to kill microorganisms. The recommended minimum exposure period for steam sterilization of wrapped healthcare supplies is 30 minutes at 121°C.^b^ Per our protocol, “Pass” is maintaining 121C for a minimum of 35 minutes. “Fail” is the failure to maintain 121°C for 35 minutes.
Sterilization	Sterilization external to packaged instruments	Sterilizer Tape	Black stripes appear if the tape has been properly exposed to steam during a sterilization run. “Pass” is when the black stripes appear. “Fail” is when the black stripes do not appear.^c^
Sterilization	Sterilization internal to packaged instruments	Indicator Strip	Strip has a “Fail” and “Pass” reading. After a sterilization run, “Pass” is equivalent to a biological kill. “Fail” is not equivalent to a biological kill.^d^
Sterilization	Sterilization inside the sterilizer	Biological Indicator Tube	After the sterilization run is completed, tube is incubated for a minimum of 10 hours. Post incubation, “Pass” means the tube is purple in color, indicating that the biological agents have been killed. “Fail” means the tube is yellow in color, indicating that the biological agents have not been killed.^e^

a. ATP is *adenosine triphosphate*, an energy-carrying molecule found in the cells of bacteria, yeast, and mold cells. Measurement of ATP documents and provides quantitative measurement to indicate the level of cleanliness and hygiene of high touch surfaces and medical instruments. See Centers for Disease Control and Prevention, Healthcare-Associated Infections, http://www.cdc.gov/hai/toolkits/Appendices-Evaluating-Environ-Cleaning.html. See also Getinge, USA. Available: http://www.getingeusa.com/life-science/consumables/contamination-monitoring-system/getinge-assure-safestep-monitoring-system/.

b. Rutala WA, Weber DJ, HICPAC. Guideline for disinfection and sterilization in healthcare facilities. Centers for Disease Control, 2008. Available: http://www.cdc.gov/hicpac/pdf/guidelines/Disinfection_Nov_2008.pdf. Temperature gauge is a geared steam gauge #72S Available: http://www.allamerican-chefsdesign.com/admin/fileuploads/product_43.pdf. Timer was electronic ‘stop watch’ on mobile telephone.

c. 3M. Available: http://www.3m.com/3M/en_US/company-us/all-3m-products/~/3M-Comply-Lead-Free-Steam-Indicator-Tape?N=5002385+8707795+8707798+8711017+8711099+8711113+8719143+3293316298&rt=rud.

d. Getinge USA. Available: http://www.getingeusa.com/healthcare/products-within/sterilization/sterility-assurance-products/chemical-sterilization-monitors/steam-sterilization-integrators/.

e. Getinge USA. Available: http://www.getingeusa.com/healthcare/consumables/sterility-assurance-products/biological-indicators/getinge-assure-accufast/.

### Decontamination

To simulate contaminated instruments arriving from the operating room, six stainless steel surgical instruments (2 mayo scissors, straight, 17.1cm; 2 halsted mosquito forceps, curved, 12.7cm; 2 backhaus towel clamps, 13.3cm. NovoSurgical) commonly used in low resources settings were contaminated in biofilm dirt. The biofilm dirt, consisting of cow liver (3.0 parts), lactose (1.0 parts), and sunflower oil (0.1 parts), was formulated to simulate the organic materials contaminating instruments and that could satisfactorily adhere, be applicable at elevated temperatures, and pose minimal sanitation and health risks to the staff. [8] The general practice was to lightly coat the instruments with the biofilm dirt, place them into a small plastic container (with a lid), and leave them sitting overnight (~12 hours; in a few cases <12 hours). To obtain an initial reading of the contamination on the instruments, we measured the bioburden using an ATP (*adenosine triphosphate*) bioluminescence device (Getinge Assure SafeStep Contamination Monitoring System). A reading of >45 on the ATP bioluminescence device indicates that the instruments were successfully contaminated. [9]

Next, the instruments were washed in the three-basin sink, with gross rinse in the first basin, a five-minute soak in enzymatic detergent (Certol ProEZ 1) and scrub with nylon brushes (KeySurgical) in the second basin, and a rinse with water in the third basin. The instruments were left to air dry in a drying rack adjacent to the third basin. Post-cleaning measurements were obtained using the ATP bioluminescence device.

### Sterilization

Immediately after decontamination, the set of six medical instruments were packaged with autoclave wrapping paper (Kimberly Clark). A plastic indicator strip (Getinge Steam Sterilization Integrator for Use in Steam Sterilizers, Part No. 61301600556) was placed into the package. The package was secured with steam indicator tape (3M or Propper), then loaded into the internal compartment of the non-electric sterilizer (WAFCO 1925X). To simulate a larger and more challenging quantity of instruments, six aluminum bars and tubes with a combined weight of 2.64 kg were added to each load. One colorimetric biological indicator tube containing *geobacillus stearothermophilus* (Getinge Assure AccuFast Biological Indicator) was placed on top of the package. Note that these three indicators, plus the temperature readings from the sterilizer itself, represent mechanical, chemical, and biological monitors of the efficacy of the steam sterilization process consistent with CDC guidelines [10].

The external compartment of the non-electric sterilizer was filled with distilled water to a 1” (2.54cm) depth. The instruments were loaded into the internal basket of the sterilizer and the sterilizer’s lid was greased and secured. The hotplate was switched on. The sterilizer was then run according to manufacturer recommendations. Once the internal temperature passed 121°C, the autoclave was manually vented for 7 minutes. The autoclave was heated to 121°C for a second time and maintained above 121°C for 35 consecutive minutes. Upon completion of this sterilization cycle, the hotplate was turned off and the sterilizer manually vented.

After cooling, the sterilizer was opened and the package removed. At this point, the operator observed whether the steam indicator tape had changed colors. The operator then opened the autoclave wrapping paper (compromising sterility for the purpose of experimental verification) and observed whether the plastic indicator strip inside displayed a pass or fail. Finally, the biological indicator tube was incubated for a minimum period of 10 hours following the manufacturer’s instructions. If the biological indicator remained purple in color, it indicated that the sterilization had been successful; if the indicator turned yellow, the bacteria had survived and the sterilization had failed.

Each cycle was followed by general cleaning and maintenance, including wiping down the sinks and flat surfaces of the sterile processing unit with cleaning solution, arrangement of sterilized medical instruments, and sweeping the container’s floor.

## Results

Table 2 reports the results of the decontamination and sterilization efficacy tests.

**Table 2:** Results of Decontamination and Sterilization Tests^a^.

DECONTAMINATION			STERILIZATION			
Pre-Decon ATP mean	Post-Decon ATP mean	Post-Decon Pass%^b^	Temperature Pass%^c^	Sterilizer Tape Pass%^d^	Indicator Strip Pass%^e^	Bioindicator Tube Pass%^f^ Pass%^f^
918.09	4.34	100	98.36	100	100	100

a. 61 trials conducted at Rice University between May 27, 2015 and August 17, 2015.

b. ATP score of <45 after decontamination.

c. 121°C for 35 minutes consecutively. In trial #44, experimenter recorded 25 minutes, instead of 35 minutes per the protocol. Note that the three other sterilize indicators made “Pass” in trial #44.

d. Stripes appear after the sterilization cycle.

e. Indicator is in the “Pass” zone after the sterilization cycle.

f. After the sterilization cycle is complete, tube incubated for >10 hours. “Pass” means tube is purple after incubation.

Decontamination was successfully achieved in each of the 61 trials. The mean initial contamination level was 709.95 ATP units, with a range of 47 to 5,324 ATP units. The post-decontamination level was a mean of 4.49 ATP units, with a range of 0 to 31 ATP units. In every trial, the post-decontamination level of ATP was well below 45, the standard cut-off for contaminated versus clean. [9]

Sterilization also was successfully achieved in each of the 61 trials using four indicators of sterilization efficacy. The recommended exposure temperature and time for sterilization, 121°C (per the sterilizer’s geared steam gauge) temperature for 35 consecutive minutes, was met in every trial, except one, when the experimenter recorded the time as only 25 minutes (note that the other three sterilization indicators all passed in this trial, suggesting that this was a recording error and not a run-time error). The autoclave indicator tape changed colors in each of the 61 trials. The plastic indicator strip finished in the “Pass” position in each of the 61 trials. Finally, the post-sterilization incubation of the biological indicator tube showed that the microorganisms had been killed in each of the 61 trials.

Our 61 successes in 61 trials give us an estimated probability of a failed decontamination and sterilization event of 0%. Using the “Rule of Three,” [11], [12] developed to estimate the confidence around an intervention, we estimate the upper 95% confidence bound for our estimate *p* is 0.04918. Thus, we are 95% confident that the probability of a failed decontamination and sterilization is less than 5%.

## Discussion about Dissemination Opportunities and Challenges

Encouraged by the solid technical results, we briefly describe several opportunities and challenges for bringing the sterile box into low resources areas. For opportunities, we identify hospitals and other healthcare facilities with needs for sterile instruments, maternal and neonatal care and oral health, post-disaster healthcare, and flexibility into other healthcare services. For challenges, we identify costs and network of partners.

### Opportunities

#### Hospitals and other healthcare facilities needing sterile instruments

The primary intention behind the creation of our sterile processing unit is to serve hospitals and other healthcare facilities that require sterile instruments but are incapable of meeting such needs. (We include a short video of how the sterile box might serve such needs in S1 Video) Healthcare facilities have significant incentives to possess sterile processing capacity as deficiencies lead to poor patient outcomes including surgical site infections, [13] extended hospital stays, [14] and even deaths. [15] Since one of the primary advantages of our sterile processing unit is that it contains its own infrastructure, i.e., electricity and water supply, it is best suited for “off-grid” healthcare facilities that perform procedures such as surgeries requiring sterile medical instruments. To single out one region of potential need, Africa has 114,578 healthcare facilities, comprised of 5,520 are district/rural hospitals, 29,770 are health centers, and 79,288 are health posts. [16], of which many are off grid.

#### Maternal and neonatal care and oral health

While sterile processing of stainless steel instruments is useful across surgical categories, maternal and neonatal care and oral health represent critical areas for sterile instruments. Infection in obstetrics is the second most common cause of maternal mortality after post-partum hemorrhage. A study in Ethiopia showed that rural women had more complicated surgical site infections compared to urban women, partially because rural women lacked access to certain emergency treatments that could be aided by sterilized instruments. [17]. In oral health, dental services are underdeveloped and mostly limited to urban areas. [18]. Many dentists do not have a way of properly sterilizing their instruments. [19] Since our unit runs a steam sterilizer, recommended by the WHO as the preferred standard of care at the district hospital level, [20] we believe that it is appropriate for preparing instruments for such procedures.

#### Post-disaster

After large-scale disasters destroy or severely damage existing healthcare facilities and supporting infrastructure, our sterile processing unit might serve to sterilize medical instruments for other post-disaster medical providers. We are aware, for example, of organizations that have introduced container-based healthcare facilities into post-tsunami Philippines to serve fairly large populations. Our sterile processing unit could complement such providers by sterilizing their medical instruments if they are unable since our unit contains its own source of water and electricity to perform such procedures.

#### Flexibility to other healthcare services

We can envision that the containerized sterile processing unit could be modified to create diagnostic labs, patient rooms, and other functions that serve the healthcare facility. For example, our 750w electric hotplate could power equipment other than the sterilizer. The inverter could support telemedicine by charging batteries of cell phones and laptop computers.

### Challenges

We briefly describe two significant challenges to the successful implementation of the sterile processing unit.

#### Costs

As previously stated, the locations most appropriate for the sterile processing unit are those without power or with unreliable power such as may be found in developing countries in rural and semi-rural districts as well as some secondary and tertiary cities. The healthcare facilities in such locations would appear to benefit from a sterile processing unit that is self-sufficient in electricity as well as water. However, even with a need for sterile instruments, the costs of providing resources towards sterile processing may be too high versus other needs. The cost of our sterile processing unit is approximately $10,000 USD, of which about one-third is the electricity infrastructure. A healthcare facility may choose to devote such investment towards other activities (such as running other equipment, i.e., refrigerators for medicines).

#### Network of partners

For the sterile box to serve the healthcare facility to its potential, it must be linked into a network of partners. [21] The healthcare facility’s management and staff (i.e., nurses in the operating room) must value quality output from sterile processing. The sterile processing unit needs to be incorporated into the other systems within the healthcare facility (and system) aimed at infection control. Although we attempted to limit their use, our sterile processing unit uses consumables such as enzymatic detergents and indicator tape and strips for quality control, and as such needs reasonable access to distribution systems for such products. Unique to the sterile box over more conventional off-grid sterile processing set-ups, is its infrastructure, such as solar PV panels and a battery system, which requires partnerships with individuals or entities (i.e., technicians, electricians) skilled in servicing such equipment. Partnerships appear indispensable to keep such interventions working to their fullest potential.

## Conclusions

Results of 61 trials of our shipping container-based sterile processing unit, the sterile box, demonstrate resoundingly its ability to decontaminate and sterilize dirty medical instruments. Passing every trial, the sterile processing unit produced successful outcomes for decontamination, as measured by the ATP bioluminescence device, and steam sterilization, as measured by temperature and time (with one exception), steam sterilizer indicator tape, plastic indicator strip, and biological indicator tube. The testing results give us confidence that the sterile processing unit performs well–the rule of three places a 95% confidence interval that the *p* = 0.04918 of a decontamination and sterilization failure–and is ready to be tested in the field.

We note opportunities and challenges exist for such a sterile processing unit going forward. Opportunities are the many hospitals and healthcare facilities desiring sterile instruments but presently unable to have them, maternal and neonatal care and oral health, post-disaster, and flexibility to host other healthcare services. Challenges are costs and finding reliable partners. Our hope is that the sterile box offers net benefits to many healthcare facilities, aiding a reduction of instrument-related surgical site infections and improving patient well-being.

## Supporting Information

S1 Appendix (DOCX)

S1 Video (AVI)
