# Supplementary material for: A Shipping Container-Based Sterile Processing Unit for Low Resources Settings
Source: PLoS One. 2016 Mar 23;11(3):e0149624. doi: 10.1371/journal.pone.0149624 (PMC4805258; doi:10.1371/journal.pone.0149624)
Supplement: S1 Appendix — (DOCX) [file pone.0149624.s001.docx]

**S1 Appendix**

**Technical Specifications of the Shipping Container-Based Sterile Processing Unit**

| **Element** | **Purpose** | **Technical Specs** |
| --- | --- | --- |
| **Container** | To act as the house of the sterile processing unit | ‘Conventional’ 20’ container made of corrugated weathering steel (‘Cor-Ten’); 6.1m length, 2.44 m wide, 2.59m height |
| **Decontamination** | To receive dirty instruments from healthcare staff. To deliver water to user to perform manual decontamination | Stainless steel table |
|  |  | Stainless steel 3-basin sink |
|  |  | Brass ball-valve |
|  |  | Drain |
|  |  | Plastic drying rack |
| **Sterilization** | To steam sterilize the instruments | Stainless steel table |
|  |  | WAFCO All-American 1925X non-electric steam sterilizer, capacity 24 liter |
|  |  | Electric hotplate |
|  |  | Small wire table |
| **Storage** | For cooling of instruments and clean and secure storage until delivery to healthcare staff | Wire rack |
|  |  | Plastic cabinet |
| **Water System** | To collect and deliver the water for decontamination | 1 55 gallon polyethylene tank. |
|  |  | 1 Ace Roto-Mold 50 gallon tank |
|  |  | Protek hand diaphragm pump |
|  |  | Assorted rubber and PVC tubes |
|  |  | Assorted ball valves and fittings. |
| **Electrical System** | To collect, store, and deliver the electricity | 4 BP Solar BP3230T PV 230w panels |
|  |  | Outback Power Charge Controller Flexmax 80 |
|  |  | 2 12V Vision Battery SCP121050 G31 (24V system) |
|  |  | Samlex Power 600w/24v inverter |
|  |  | Mounting racks |
|  |  | Cables |
